# Activities of the peptidyl transferase center of ribosomes lacking protein L27

**DOI:** 10.1261/rna.053330.115

**Published:** 2015-12

**Authors:** Cristina Maracci, Ingo Wohlgemuth, Marina V. Rodnina

**Affiliations:** Department of Physical Biochemistry, Max Planck Institute for Biophysical Chemistry, 37077 Goettingen, Germany

**Keywords:** ribosome, translation, peptide-bond formation, tRNA, ribozyme

## Abstract

The ribosome is the molecular machine responsible for protein synthesis in all living organisms. Its catalytic core, the peptidyl transferase center (PTC), is built of rRNA, although several proteins reach close to the inner rRNA shell. In the *Escherichia coli* ribosome, the flexible N-terminal tail of the ribosomal protein L27 contacts the A- and P-site tRNA. Based on computer simulations of the PTC and on previous biochemical evidence, the N-terminal α-amino group of L27 was suggested to take part in the peptidyl-transfer reaction. However, the contribution of this group to catalysis has not been tested experimentally. Here we investigate the role of L27 in peptide-bond formation using fast kinetics approaches. We show that the rate of peptide-bond formation at physiological pH, both with aminoacyl-tRNA or with the substrate analog puromycin, is independent of the presence of L27; furthermore, translation of natural mRNAs is only marginally affected in the absence of L27. The pH dependence of the puromycin reaction is unaltered in the absence of L27, indicating that the N-terminal α-amine is not the ionizing group taking part in catalysis. Likewise, L27 is not required for the peptidyl-tRNA hydrolysis during termination. Thus, apart from the known effect on subunit association, which most likely explains the phenotype of the deletion strains, L27 does not appear to be a key player in the core mechanism of peptide-bond formation on the ribosome.

## INTRODUCTION

In all living organisms, the ribosome catalyzes the sequential polymerization of amino acids into functional proteins. In each round of translation elongation, the ribosome selects an aminoacyl-tRNA (aa-tRNA) corresponding to the mRNA codon presented in the A site. After accommodation of the 3′ end of the A-site tRNA in the peptidyl transferase center (PTC) of the ribosome, the amino group of the aa-tRNA nucleophillically attacks the ester of the peptidyl-tRNA in the P site leading to the formation of a peptide bond and the transfer of the peptidyl chain onto the A-site tRNA. The PTC resides in the large subunit of the ribosome (50S in bacteria) and is constituted mainly by rRNA, which is thought to be responsible for the catalytic activity. However, crystal structures of the *Thermus thermophilus* ribosome in complex with tRNAs (Selmer et al. 2006; Voorhees et al. 2009) showed that protein L27, which is conserved in prokaryotes, extends far enough into the PTC to potentially contribute to catalysis. L27 is a small protein located at the base of the central protuberance of the 50S subunit. Its long, conserved N-terminal tail protrudes into the PTC, where it was reported to interact with both A-site and P-site tRNAs (Wower et al. 2000; Maguire et al. 2005; Voorhees et al. 2009; Jenner et al. 2010).

Peptide-bond formation on the ribosome is for most aa-tRNAs rate-limited by their accommodation into the A site (Pape et al. 1998; Wohlgemuth et al. 2010; Johansson et al. 2011). Therefore, pre-steady state kinetic studies that aimed at determining the reaction mechanism often utilized aa-tRNA analogs, such as puromycin (Pmn) or its derivatives (e.g., C-Pmn [Katunin et al. 2002; Okuda et al. 2005; Brunelle et al. 2006; Wohlgemuth et al. 2006; Beringer and Rodnina 2007]). When Pmn is used as substrate, the peptidyl-transfer (PT) reaction shows a pronounced pH dependence with two ionizing groups, indicating that besides the amino group of the substrate (6.9 for Pmn [Katunin et al. 2002]), another ionizing group, with a pKa of ∼7.5, contributes to catalysis (Katunin et al. 2002; Beringer and Rodnina 2007). Based on computer simulations of the pre- and post-PT state of the ribosome, this group has been proposed to be the N-terminal amine of L27 (Trobro and Åqvist 2008; Xiao and Wang 2012). The absence of L27 is thus predicted to alter the pH dependence of PT; however, the contribution of this ionizable group to the PT reaction has not been experimentally tested so far.

Early biochemical data indicated a functional role for L27 in the PT activity. *Escherichia coli* strains in which L27 was deleted grew six times slower than the wild type (wt), and ribosomes lacking L27 were found to carry sub-stoichiometric amounts of L16, L21, and L20. The PT activity of ΔL27 ribosomes was slightly reduced, both in the presence of Pmn or native A-site substrate (Phe-tRNA^Phe^) (Wower et al. 1998). Truncation of the N-terminal tail of L27 revealed that the absence of as few as the first three residues reduces the PT activity to the level of ΔL27 ribosomes (Maguire et al. 2005). smFRET data indicated that the main function of L27 might be to stabilize the tRNAs on the ribosome (Wang and Xiao 2012; Xiao and Wang 2012), which for some amino acids may contribute to the acceleration of the PT reaction. Furthermore, the α-amino group of L27 was recently suggested to take part in proton transfer during the PT reaction, assisting the 5′-phosphate oxygen of the A-site A76 in the deprotonation of the nucleophile in the transition state (Polikanov et al. 2014). Although L27 is not present in Archaea and Eukaryotes, another ribosomal protein, the archaeal L10e and its eukaryotic homolog RPL10, extends a loop in the same position as the L27 tail (Armache et al. 2010); mutations in this conserved loop are lethal (Hofer et al. 2007). Validation of the role of L27 in the PT reaction for all or perhaps only certain incoming amino acids would, however, require detailed biochemical and kinetic studies. Here we use rapid kinetics methods to precisely quantify the contribution of L27 on the activity of the PTC.

## RESULTS AND DISCUSSION

**FIGURE 1.**
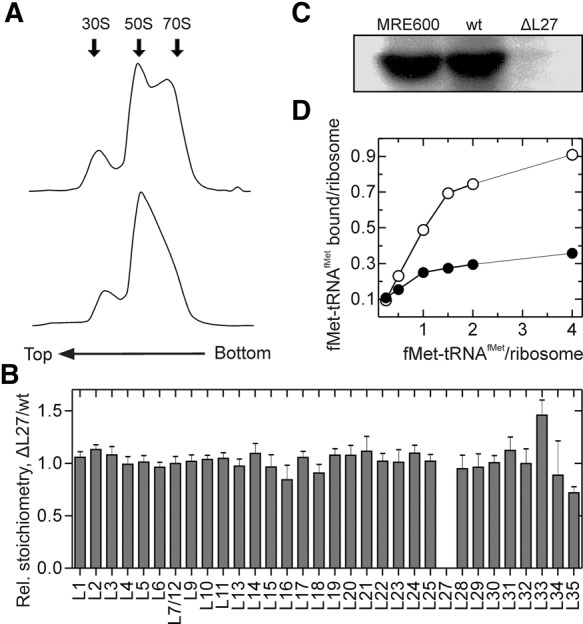
Characterization of ΔL27 ribosomes. (*A*) Sucrose-gradient centrifugation profile of wt (*upper* panel) and ΔL27 (*bottom* panel) ribosomes at 5 mM Mg^2+^. (*B*) Quantification of ribosomal proteins by mass spectrometry. The ratio of the average protein concentrations (as defined by label-free quantification) ΔL27/wt was plotted. Error bars represent the standard deviation of four technical replicates. (*C*) Western blot of ribosomal proteins from MRE600, wt, and ΔL27 ribosomes using anti-L27 antibody. (*D*) Determination of the active concentration of ΔL27 ribosomes. The extent of initiation was determined by the radioactivity retained on nitrocellulose filters after incubation of wt (open circles) or ΔL27 (closed circles) ribosomes with mRNA, initiation factors, GTP, and increasing concentrations of f[^3^H]Met-tRNA^fMet^.

We purified ribosomes from the *E. coli* strain lacking the gene *rpmA* encoding L27 (IW312 strain; hereafter referred to as ΔL27) and from the same strain expressing L27 from a plasmid (IW312 + pPOTA1:*rpmA*; hereafter referred to as wt). Ribosome profiles obtained by sucrose-gradient centrifugation showed the absence of a pronounced 70S peak in the ΔL27 strain, indicating that most ribosomes are dissociated into the subunits at 5 mM Mg^2+^ (Fig. 1A). In accordance with earlier results of sucrose-gradient analysis (Wower et al. 1998), impaired subunit association might explain the slow growth phenotype of this strain. We thus collected fractions containing a mixture of 50S and 70S and confirmed the absence of L27 by quantitative mass spectrometry (Fig. 1B) and Western blotting (Fig. 1C). The active ribosome concentration was determined by preparing 70S initiation complexes in the presence of mRNA, initiation factors, and increasing concentration of f[^3^H]Met-tRNA^fMet^ (Fig. 1D). More than 90% of the wt ribosomes formed stable complexes with fMet-tRNA^fMet^, whereas ΔL27 ribosomes initiated to only ∼40%, indicating that the preparation contains significant portion of free 50S subunits and/or incomplete and inactive 70S ribosomes.

**FIGURE 2.**
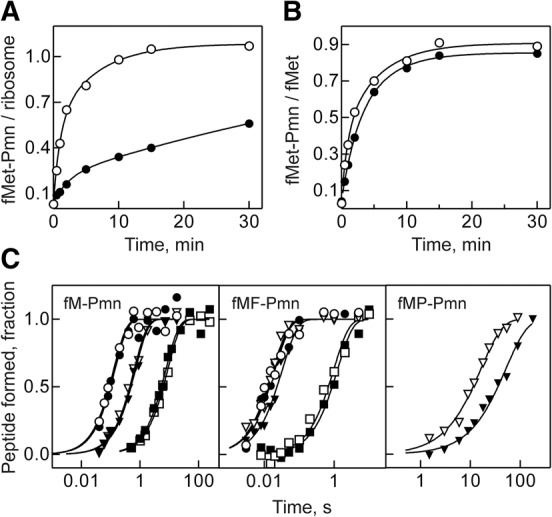
Peptide-bond formation with Pmn as A-site substrate. Unpurified (*A*) or sucrose gradient-purified (*B*) ICs prepared with wt (open circles) or ΔL27 (closed circles) ribosomes (0.25 µM) were mixed with Pmn (1 µM), and the extent of dipeptide reaction was monitored over time. Solid lines represent the results of exponential fitting (see Materials and Methods). (*C*) Time courses of fMet-Pmn (*left* panel), fMetPhe-Pmn (*middle* panel), and fMetPro-Pmn (*right* panel) formation upon mixing of wt (open symbols) and ΔL27 (closed symbols) IC (50 nM) with high concentrations of Pmn (2.5–10 mM) at pH 6.5 (squares), 7.5 (triangles), and 8.5 (circles). Time courses were normalized for the extent of the reaction to facilitate visual inspection. Solid lines represent the results of exponential fitting of the time points. Rates of the fMet-Pmn reaction for wt and ΔL27 ribosomes, respectively, were 0.11 ± 0.1 and 0.13 ± 0.1 sec^−1^ at pH 6.5; 1.6 ± 0.1 and 1.4 ± 0.1 sec^−1^ at pH 7.5; and 7.8 ± 1.7 and 7.1 ± 1.9 sec^−1^ at pH 8.5. Rates of the fMetPhe-Pmn for wt and ΔL27 ribosomes, respectively, were 1.0 ± 0.2 and 0.8 ± 0.1 sec^−1^ at pH 6.5; 55 ± 6 and 30 ± 3 sec^−1^ at pH 7.5; and 55 ± 10 and 49 ± 10 sec^−1^ at pH 8.5. Rates of the fMetPro-Pmn reaction at pH 7.5 were 0.06 ± 0.01 and 0.02 ± 0.01 sec^−1^ for wt and ΔL27 ribosome, respectively.

Previous reports indicated that in the presence of low concentrations of Pmn, formation of fMet-Pmn is impaired on ribosomes lacking L27 (Maguire et al. 2005). Because Pmn concentrations used in those experiments (1 µM) were far below the K_M_ value for the ribosome–Pmn complex (∼1–30 mM, depending on the tRNA in the P site) (Youngman et al. 2004; Beringer et al. 2005; Wohlgemuth et al. 2008), the observed decrease in the kinetics of the Pmn reaction might be due to a Pmn binding defect, rather than the reduced PT rate. To distinguish between the K_M_ and k_cat_ effects, we first reproduced the previous studies using fMet-tRNA^fMet^ and Pmn as P- and A-site substrates. Indeed, the extent of fMet-Pmn formation with ΔL27 ribosomes appeared lower compared to the wt (Fig. 2A); however, when the amount of active ribosomes was taken into account, it turned out that all ribosomes active in initiation were also active in the PT reaction (Fig. 2A). To verify this result, we repeated the experiment with initiation complexes (ICs) purified through a sucrose cushion; as determined by nitrocellulose filtration, >50% of the IC formed with ΔL27 ribosomes dissociated upon purification (not shown), indicating that binding of fMet-tRNA^fMet^ was somewhat weaker on the mutant ribosomes. The active concentration of the remaining stable complexes was calculated from the amount of f[^3^H]Met-tRNA^fMet^ bound. These remaining ribosome complexes showed identical rates of peptide-bond formation (Fig. 2B), which shows that ΔL27 ribosomes do not have a PT defect.

The ribosome group presumably involved in the PT reaction with Pmn has not been identified. If the N-terminal α-amine of L27 takes part in catalysis, one would expect the pH dependence of the Pmn reaction to change in the absence of L27. To test this hypothesis, we compared the kinetics of fMet-Pmn formation at different pH values using ΔL27 and wt ribosomes. This time, we determined the maximal rate of peptide-bond formation at high concentrations of Pmn (Fig. 2C). As expected, the rate of fMet-Pmn formation depends on pH. However, at a given pH, the rates of the reaction were identical on the wt and ΔL27 ribosomes. This finding indicates that not only the PT activity at a defined pH, but also the overall rate/pH profile is not altered in the absence of L27, which further strengthens the notion that the protein itself is completely dispensable for this reaction. A similar result was obtained when we followed the Pmn reaction on post-translocation complexes carrying an fMetPhe-tRNA^Phe^ in the P site at different pH values (Fig. 2C). Also in this case the rate of fMetPhe-Pmn tripeptide formation was independent of the presence of L27. To check whether L27 might be more important for less reactive substrates such as proline (Wohlgemuth et al. 2008), which causes ribosomal stalling when more than two prolines have to be incorporated into the peptide (Doerfel et al. 2013; Ude et al. 2013), we checked the Pmn reaction with post-translocation complexes carrying a fMetPro-tRNA^Pro^ in the P site. In this case, the rate of peptide-bond formation was slightly decreased in the absence of L27 (0.02 sec^−1^, compared with the rate of 0.06 sec^−1^ obtained with wt ribosomes) (Fig. 2C).

**FIGURE 3.**
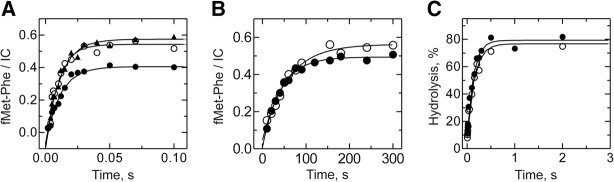
Reactions with natural substrates. (*A*) PT with cognate aa-tRNA was measured upon mixing wt (open circles), L27Δ1-6 (closed triangles), or ΔL27 (closed circles) ICs (50 nM) containing fMet-tRNA^fMet^ in the P site and a UUC codon in the A site with TC-Phe (10 µM). Solid lines represent the results of exponential fitting of the time points, which yielded the rates of 110 ± 13, 90 ± 9, and 90 ± 8 sec^−1^ for wt, L27Δ1-6, and ΔL27 ribosomes, respectively. (*B*) PT with near cognate aa-tRNA was measured as in *A*, using ICs with a CUC codon in the A site. Exponential fitting (solid lines) yielded the rates of 0.017 ± 0.002 and 0.024 ± 0.002 sec^−1^ for wt and ΔL27 ribosomes, respectively. (*C*) Peptide release was measured upon mixing wt (open circles) or ΔL27 (closed circles) ICs (75 nM) programmed on a UAA codon with RF2 (1.5 µM). The rates, determined by exponential fitting (solid lines), were 6.6 ± 0.6 and 7.6 ± 0.8 sec^−1^ for wt and ΔL27 ribosomes, respectively.

Although L27 seems to be dispensable for PT with A-site tRNA analogs, it might still play a role in the presence of full-length aa-tRNAs. We thus determined the rate of fMet-Phe formation in the absence of L27. ICs were mixed with saturating concentrations of EF-Tu–GTP–Phe-tRNA^Phe^ ternary complex (TC-Phe) (Fig. 3A). The extent of dipeptide formation was lower on ΔL27 ribosomes (40%) compared with wt ribosomes (60%), as previously observed (Wower et al. 1998). However, the rate was very similar to the wt control (90 sec^−1^ and 110 sec^−1^ for ΔL27 and wt, respectively). To investigate the origin of this small difference, we also measured the rate of dipeptide formation on ribosomes carrying an N-terminal truncated version of L27 (L27 Δ1-6). The dipeptide rate obtained with these ribosomes, 90 sec^−1^, indicates that the small difference is due solely to the presence of the first six residues of L27 (Fig. 3A).

Apart from the proposed direct participation in the PT mechanism, the L27 tail might be involved in cognate aa-tRNA selection. In fact, the crystal structure of the *T. thermophilus* ribosome in complex with a near-cognate ternary complex shows the N-terminal tail of L27 in a different conformation as compared to the cognate tRNA (Jenner et al. 2010). We reasoned that if L27 is involved in tRNA selection, PT to a near-cognate aa-tRNA would be affected by the absence of L27. To test this hyphothesis, we reacted ribosomes carrying fMet-tRNA^fMet^ in the P site and a CUC codon in the A site (coding for leucine) with an excess of the near cognate Phe-tRNA^Phe^–EF-Tu–GTP (Fig. 3B). In this case, the extent and the rate of the PT reaction were essentially identical (rates of 0.014 and 0.019 sec^−1^ for wt and ΔL27 ribosomes, respectively). In summary, our data argue against any significant involvement of L27 in the mechanism of peptide-bond formation.

In addition to the PT reaction, the PTC catalyzes the hydrolysis of the peptidyl tRNA during translation termination. This prompted us to measure the impact of L27 in this reaction. We prepared pre-termination complexes with fMet-tRNA^fMet^ in the P site and a UAA stop codon in the A site and followed the kinetics of peptide release in the presence of saturating concentrations of RF2. Also in this case, no difference was observed between the wt and ΔL27 ribosomes (Fig. 3C). The rate of peptide release (∼7 sec^−1^) was in agreement with that previously determined using wt (MRE600) ribosomes (Kuhlenkoetter et al. 2011; Indrisiunaite et al. 2015).

**FIGURE 4.**
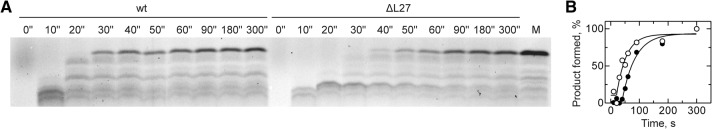
Single-round translation of CspA mRNA. (*A*) Time courses of in vitro translation using wt and ΔL27 ribosomes. (M) Marker, translation product obtained with wt MRE600 ribosomes. (*B*) Kinetics of the translation by wt (open circles) and ΔL27 (closed circles) ribosomes, as obtained by densitometry of the full-length CspA bands shown in *A*. The last point in each time course was set to 100%.

To investigate the importance of L27 for the steps of the elongation cycle that are not reflected in our model assays (e.g., translocation, tRNA competition) or for context-dependent effects on peptide-bond formation, we translated a full-length natural mRNA with ΔL27 ribosomes, using the mRNA coding for the *E. coli* CspA as a model (Rudorf et al. 2014). The time courses of translation (Fig. 4A) show that the mutant ribosomes translate the mRNA as efficiently as the wt ribosomes and without significant pausing, confirming that deletion of L27 does not have adverse effects on incorporation of amino acids other than Phe that was tested above. The average elongation rate per codon, as determined by fitting of the intensity change of the full-length product, was 1.3 aa sec^−1^ for the wt and 0.8 aa sec^−1^ for the ΔL27 ribosomes (Fig. 4B).

Taken together, our data strongly argue against an important contribution of L27 to the core mechanism of peptide-bond formation or peptidyl-tRNA hydrolysis at the PTC. Previously reported phenotypes of the mutant ribosomes can, in our opinion, be explained by the fact that the absence of L27 impairs the 50S subunit assembly and subunit association, thereby yielding a nonhomogeneous population of active and inactive ribosomes inside the cell. Indeed, the major phenotype we observed is an impaired 70S ribosome formation, which could explain the growth phenotype and the low yield of translation initiation. One small additional effect may be related to the stabilization of the substrates on the ribosome by the N-terminal tail of L27, as suggested by the subtle kinetic effects of L27 on fMetPhe formation and overall translation.

There are currently two main suggestions for the mechanism of ribosomal peptide-bond formation, the so-called proton shuttle (Trobro and Åqvist 2005) and the proton wire (Polikanov et al. 2014) models. Both models are consistent with the experimental data available so far, including the pH/rate profiles for different substrates, mutational analysis, and KIE and KSIE measurements (Katunin et al. 2002; Youngman et al. 2004; Beringer and Rodnina 2007; Lang et al. 2008; Wohlgemuth et al. 2008; Hiller et al. 2011; Kuhlenkoetter et al. 2011; Zaher et al. 2011). Both models suggest that the proton from the attacking amine is transferred to the leaving group through the 2′-OH of the P-site tRNA A76; however, the impact of L27 and the exact path of proton transfer is different (we note that at a time when the proton shuttle model was suggested, the positions of the water molecules in the catalytic site were not known with precision). Although L27 does not play a role in the proton shuttle model, the wire model (Polikanov et al. 2014) suggests a contribution of the N-terminal amine of L27 in restricting the access of water molecules by closing the reaction pocket, thereby delaying the loss of a proton from a catalytic water molecule to the bulk solvent. The wire model would predict that the absence of the N terminus of L27 should alter both the rates of intermediate formation and the conversion of the intermediate into products, which is clearly not consistent with the present experiments. However, we cannot exclude that in the absence of L27, some other group (protein, rRNA, or water) takes over the functions of L27 (e.g., closes the reaction pocket). Alternatively, it is also possible that although the wire model is correct as to the path for proton transfer, L27 does not play the proposed role even though it is located in the vicinity. Finally, both pathways—the shuttle and the wire—may turn out to be isoenergetic; in the absence of L27, the ribosome may favor the shuttle mechanism that does not involve L27.

## MATERIALS AND METHODS

### Strains, chemicals, and buffers

*E. coli* strains IW312 (ΔL27) and IW312 expressing the full-length L27 (wt) or its N-terminally truncated version (L27 Δ1-6) from the plasmid pPOT1AE were a kind gift from Robert Zimmermann (University of Massachusetts Amherst) (Maguire et al. 2005). Chemicals were from Roche Molecular Biochemicals, Sigma-Aldrich, or Merck. Radioactive compounds were from Hartmann Analytic. MNV mRNA (5′-GGCAAGGAGGUAAAUA*AUGNNNACGAUU*-3′, in which the coding sequence is in italics and the A-site codon (underlined) is NNN (UUC coding for Phe, CUC coding for Leu, CCG coding for Pro, and UAA as the stop codon), was purchased from IBA. Buffer A: 50 mM Tris–HCl, pH 7.5, 70 mM NH_4_Cl, 30 mM KCl, and 7 mM MgCl_2_. Buffer B (HiFi): 50 mM Tris–HCl, pH 7.5, 70 mM NH_4_Cl, 30 mM KCl, 3.5 mM MgCl_2_, 0.5 mM spermidine, 8 mM putrescine, and 2 mM DTT. The pH of buffers and Pmn stocks was adjusted to 6.5, 7.5, or 8.5 at 37°C.

### Ribosomes, initiation, and ternary complexes

For ribosome preparation, cells were grown in LB medium supplied with 50 µg/mL kanamycin, 1 mM ITPG, and, where appropriate, 50 µg/mL ampicillin, as described in Maguire et al. (2005). Ribosomes, initiation factors, EF-Tu, EF-G, tRNAs, and RF2 were prepared as previously described (Rodnina et al. 1995; Gromadski and Rodnina 2004; Kuhlenkoetter et al. 2011). Ternary complex EF-Tu–GTP–Phe-tRNA^Phe^ was prepared by incubating EF-Tu (40 µM), GTP (1 mM), phosphoenol pyruvate (3 mM), pyruvate kinase (0.05 mg/mL), and [^14^C]Phe-tRNA^Phe^ or [^14^C]Pro-tRNA^Pro^ (20 µM) in buffer A. Initiation complexes (ICs) were prepared by incubating ribosomes (1 µM) with mRNA (3 µM), initiation factors (1.5 µM each), f[^3^H]Met-tRNA^fMet^ (2 µM), and GTP (1 mM) in buffer A for 30 min at 37°C. Post-translocation complexes carrying fMetPhe-tRNA^Phe^ or fMetPro-tRNA^Pro^ in the P site were formed by reacting ICs with a twofold excess of the suitable TC and catalytic amounts of EF-G for 5 min at 37°C. Ribosome complexes were purified by centrifugation through sucrose cushions (1.1 M sucrose in buffer A) at 259,000*g* for 2 h. Pellets were dissolved in buffer A and stored at −80°C in small aliquots.

### Western blot

Twenty-five picomoles of MRE600, wt or ΔL27 70S ribosomes were digested for 1 h at 37°C with 0.1 µg/µL Benzonase RNase (Sigma-Aldrich) before loading on an 18% acrylamide SDS-PAGE gel. The presence of L27 was determined with the use of polyclonal antibodies against L27.

### Mass spectrometry

One hundred picomoles of wt and ΔL27 ribosomes were proteolyzed with trypsin and analyzed by LC-ESI MS/MS as described (Davydov et al. 2013). Thermo RAW files were processed with MaxQuant (1.5.2.8) against a Uniprot *E. coli* (K12) database. 50S subunit proteins were quantified by intensity-based label-free quantification using MaxQuant Label Free Quantification (LFQ) values (Cox et al. 2014).

### Kinetics experiments

All experiments were carried out at 37°C, if not stated otherwise. The formation of f[^3^H]Met-Pmn and of f[^3^H]Met[^14^C]Phe/Pro-Pmn was measured upon rapid mixing of equal volumes (14 µL) of ICs (50 nM; final concentrations are reported throughout the paper) and Pmn (2.5–10 mM) in a quench-flow apparatus (RQF-3, KinTek Corporation). Samples were quenched with 25% formic acid and f[^3^H]Met-Pmn was released upon addition of 500 µL of 1.5 M sodium acetate (pH 4.5) saturated with MgSO_4_ and extracted in 750 µL ethyl acetate. Radioactivity in 500 µL of the organic phase was counted. Alternatively, samples were quenched with 0.5 M KOH, and f[^3^H]Met[^14^C]Phe/Pro-Pmn peptides were separated by reverse-phase HPLC (see below). For reactions with aa-tRNAs, ICs (50 nM) were mixed in the quench-flow with a large excess of the ternary complex EF-Tu–GTP–Phe-tRNA^Phe^ (10 µM). Samples were quenched with 0.5 M KOH, hydrolyzed for 30 min at 37°C, and neutralized with one-tenth volume of glacial acetic acid. Dipeptides were analyzed by HPLC according to Katunin et al. (2002). Radioactivity in the eluate was counted after addition of Lumasafe Plus scintillation cocktail (PerkinElmer). Time courses were fitted to an exponential function, *F* = *F*_∞_ + *A* × exp(−*k*_app_ × *t*), with a time constant, *k*_app_, the amplitude of the signal change, *A*, the final signal, *F*_∞_, and the fluorescence signal at time *t*, *F*. For fitting of the time courses shown in Figure 2A,B, an additional exponential term was included. Calculations were performed using Prism (GraphPad software). The rates are reported ± SEM.

### In vitro translation

Single-round translation of the full-length CspA mRNA (70 aa) was performed according to Doerfel et al. (2013) and Rudorf et al. (2014). Wt and ΔL27 ICs were prepared as described above using Bodipy-FL-Met-tRNA^fMet^. Translation was initiated by mixing IC (20 nM) with a translation mix containing EF-Tu (39 µM), total aa-tRNA (19.3 µM), EF-G (2 µM), GTP (1 mM), phosphoenol pyruvate (1.3 mM), and pyruvate kinase (0.1 mg/mL) at 37°C in buffer B. The translation product was separated on a 16.5% Tris–tricine–PAGE (Schägger and von Jagow 1987) and visualized by the fluorescent reporter at the N terminus of the peptides. The intensity of the full-length product was quantified with ImageJ. Average translation rates per codon were determined by fitting with a delay phase followed an exponential function (GraphPad software).
